# Regulators of clonal hematopoiesis and physiological consequences of this condition

**DOI:** 10.20517/jca.2023.39

**Published:** 2023-12-31

**Authors:** Eunbee Park, Megan A. Evans, Kenneth Walsh

**Affiliations:** 1 Department of Biochemistry and Molecular Genetics, University of Virginia School of Medicine, Charlottesville, VA 22908, USA.; 2 Hematovascular Biology Center, Robert M. Berne Cardiovascular Research Center, University of Virginia School of Medicine, Charlottesville, VA 22908, USA.

**Keywords:** Clonal hematopoiesis, aging, inflammation, genotoxic stress

## Abstract

Clonal hematopoiesis (CH) is a prevalent condition that results from somatic mutations in hematopoietic stem cells. When these mutations occur in “driver” genes, they can potentially confer fitness advantages to the affected cells, leading to a clonal expansion. While most clonal expansions of mutant cells are generally considered to be asymptomatic since they do not impact overall blood cell numbers, CH carriers face long-term risks of all-cause mortality and age-associated diseases, including cardiovascular disease and hematological malignancies. While considerable research has focused on understanding the association between CH and these diseases, less attention has been given to exploring the regulatory factors that contribute to the expansion of the driver gene clone. This review focuses on the association between environmental stressors and inherited genetic risk factors in the context of CH development. A better understanding of how these stressors impact CH development will facilitate mechanistic studies and potentially lead to new therapeutic avenues to treat individuals with this condition.

## INTRODUCTION

Hematopoietic stem cells (HSCs) play a pivotal role in the dynamic process responsible for generating various blood cell lineages. These cells engage in self-renewal and differentiation to maintain the equilibrium of the hematopoietic system. Unlike most somatic cells, HSCs undergo infrequent divisions, a protective mechanism aimed at minimizing the risk of DNA damage caused by replication and oxidative stress. However, despite their low division rate, HSCs can accumulate somatic mutations throughout their lifespan. These mutations result in genetic variations that differ from germline cells and are subsequently passed on to their progeny blood cells. While many of these mutations have negligible biological consequences, certain cancer-related mutations arising in HSCs can confer fitness advantages. These mutations can cause clonal expansions by promoting HSC self-renewal at the expense of differentiated HSCs, reducing cell death, or a combination of both. This phenomenon is referred to as clonal hematopoiesis (CH)^[1]^. Somatic mutations can manifest as genetic alterations, including single nucleotide variants, small insertions or deletions (indels), and large-scale chromosomal alterations including deletions, duplications, and copy-neutral loss of heterozygosity^[2]^. While mosaic chromosomal alterations, such as loss of the Y chromosome, have been shown to alter immune cell function and contribute to age-associated disease^[3–7]^, this review will focus on the experimentally defined “driver” gene loci that acquire somatic mutations and give rise to clonal events.

Within the realm of CH, somatic mutations accumulate in the hematopoietic system over the course of an individual’s lifetime, yet the individual typically does not develop blood cancer or other overt hematologic disorders^[8,9]^. The size of the expanded clone assumes considerable significance from a biological standpoint. The advent of next-generation sequencing (NGS), and in particular error-corrected NGS, has revolutionized the detection and characterization of mutant clones within peripheral blood. Based on NGS analyses, a variant allele frequency (VAF) threshold of 2%, which corresponds to 4% of blood cells harboring heterozygous mutations, has been suggested as a meaningful benchmark for establishing correlations with clinically significant outcomes^[10]^. This threshold is a pivotal component within the analytical framework of NGS sequencing methods, including targeted sequencing, whole-exome, or whole-genome sequencing, which are commonly utilized for the detection of clonal hematopoiesis of indeterminate potential (CHIP). However, it is important to acknowledge that as sequencing technology has advanced, clones with VAF values below 2% have been found to be clinically significant^[11–13]^. Thus, the existing criteria for CHIP require ongoing reassessment of accuracy and relevance.

**Figure 1.**
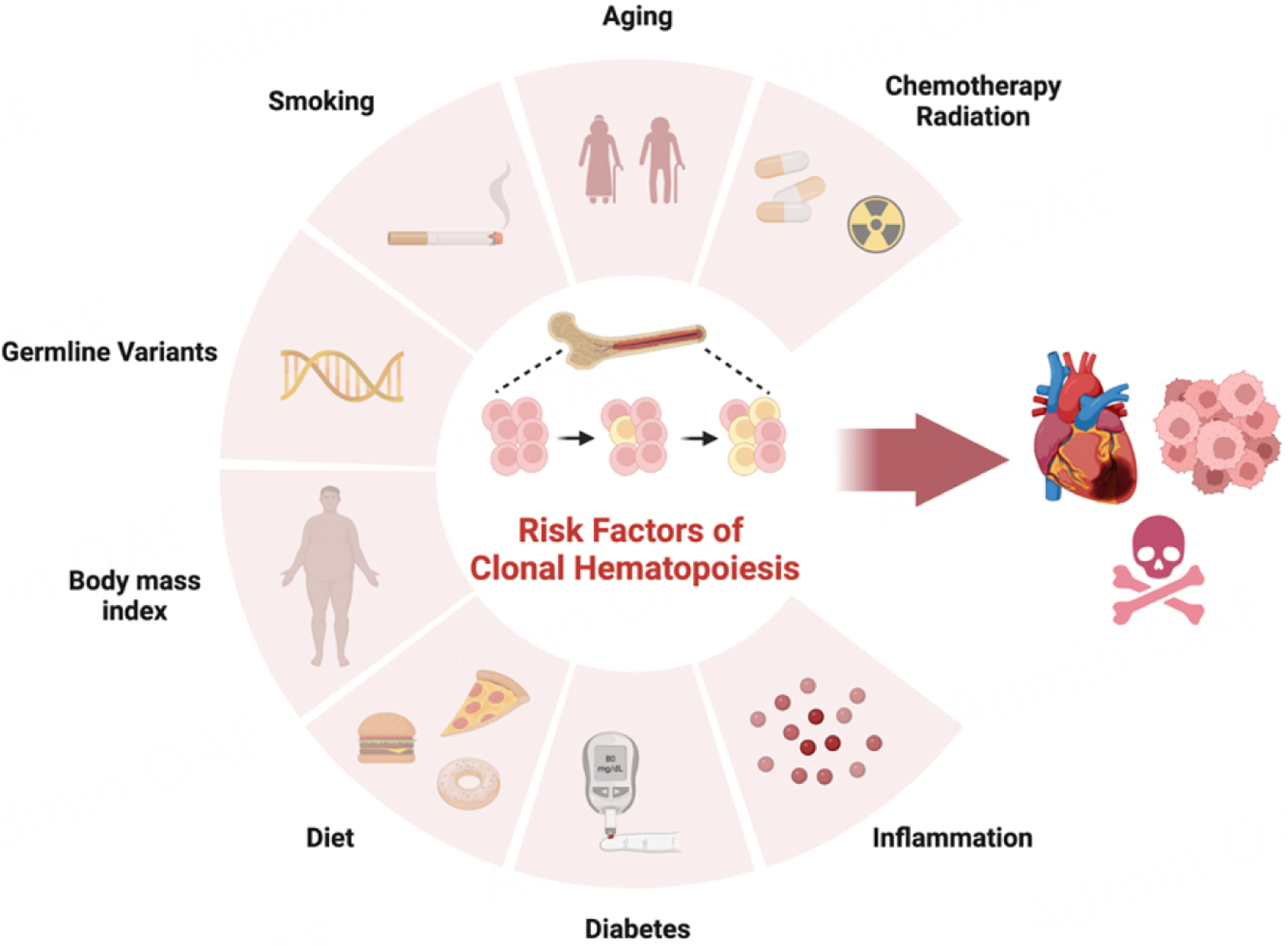
Representation of the risk factors that contribute to the expansion of CH mutant clones. The identified risk factors encompass a range of conditions and exposures, including aging, chemotherapy and radiation treatments, inflammatory processes, germline variants, and lifestyle factors such as smoking, body mass index, diet, and diabetes. The presence of these CH clones has been associated with substantial health risks, including the development of multiple age-associated diseases, including cardiovascular disease and hematological malignancies, and with increased all-cause mortality. The graphical representation of these risk factors has been generated using BioRender.com.

CH generally encompasses cases where individuals do not display conventional diagnostic indicators of blood malignancy, such as cytopenia or abnormal blood cell counts^[14]^. However, the mutations occurring in CH-driver genes have the potential to induce clonal instability and elevate the risk of hematological neoplasia^[8,9,14–17]^. Furthermore, the effect of CH extends beyond its pre-neoplastic phenotype. The clonal expansion of HSCs in CH has been found to lead to a myriad of consequences that surpass neoplastic transformation, including an elevated risk of various noncancerous age-related diseases and mortality^[1,8,16,18]^. These compelling findings have led to inquiries into the intricate mechanisms governing the selection and clonal expansion of these driver genes, thereby contributing to our understanding of the induction of pathogenicity caused by CH [Figure 1].

## EPIDEMIOLOGY: CH, CARDIOVASCULAR DISEASE AND OTHER AGE-RELATED DISEASES

The association between CH and all-cause mortality has sparked considerable interest within the scientific community. Earlier investigations conducted by Jaiswal *et al.* and Genovese *et al.* involved longitudinal studies that utilized whole-exome sequencing of DNA samples derived from peripheral blood cells^[8,9]^. These studies encompassed a substantial cohort of over 29,000 individuals without hematological malignancies. As expected from earlier work, both studies observed an age-dependent increase in the prevalence of CH-driver mutations. Remarkably, individuals with CH exhibited a significant reduction in overall survival during the follow-up period. Subsequently, Zink *et al.* corroborated these results by using whole-genome sequencing on a population of 11,262 Icelanders without hematological malignancy, and once again demonstrated a significant association between CH and higher rates of all-cause mortality^[16]^.

While the higher prevalence of hematological cancers is expected due to the early involvement of driver gene mutations in hematological malignancy progression, the relatively low frequency of hematological malignancies in the general population cannot fully account for the observed elevation in all-cause mortality in individuals with CH. Notably, Jaiswal *et al.* reported a solitary instance of mortality directly attributed to a hematological neoplasm within their study cohort of more than 5,000 individuals^[8]^. Therefore, a secondary analysis was undertaken to understand the relationship between CH and its impact on disease outcomes, with a specific focus on its association with cardiovascular disease (CVD)-related mortality^[8]^. This investigation revealed a considerably elevated risk of developing coronary heart disease (hazard ratio of 2.0) and ischemic stroke (hazard ratio of 2.6) among individuals with CH. Importantly, these associations remained significant even after adjusting for confounding factors such as age, sex, type 2 diabetes, systolic blood pressure, and body mass index.

To further validate their findings, Jaiswal *et al.* performed whole-exome sequencing on several case-control cohorts comprising 8,255 individuals^[18]^. The results of this study reinforced the association between CH and adverse cardiovascular outcomes. Specifically, individuals with CH exhibited a 1.9-fold increased risk of coronary heart disease, a 3.3-fold increased risk of coronary artery calcification, and a 4.0-fold increased risk of early-onset myocardial infarction. These compelling findings underscore the significant impact of CH on cardiovascular health. Moreover, in addition to the seminal studies mentioned earlier, several recent investigations have reported further associations between CH and various forms of CVD, including heart failure and peripheral artery disease^[12,19–30]^. In many instances, evidence of causality and mechanism has been provided by studies in model organisms^[18,24,30–35]^. Collectively, these studies contribute to a growing body of evidence linking CH to a broad spectrum of cardiovascular disorders.

In addition to its well-established association with CVD, CH has also been implicated in a range of other age-related diseases, and it is important to briefly highlight the diverse spectrum of diseases linked to this phenomenon. CH has been associated with an increased risk of various conditions, including chronic obstructive pulmonary disease^[36,37]^, chronic kidney disease^[38,39]^, diabetes and obesity^[8,40]^, chronic liver disease^[41]^, osteoporosis^[42]^, gout^[43]^, early menopause^[20]^, infectious diseases^[44–46]^, autoimmune diseases^[47–49]^, epigenetic aging^[50,51]^, and solid tumors^[52,53]^. A comprehensive review of these disease associations can be found in Evans and Walsh^[1]^. The observed associations between CH and its diverse health consequences emphasize the significance of understanding the causes of this condition and how to mitigate these effects.

It is noteworthy that nearly every individual carries detectable clonal mutations in the hematopoietic system by the age of 50, but these clones are typically well below the 2% VAF threshold of CHIP^[54]^. While these clones can increase in size as individuals reach advanced age, it is important to highlight that CH expansion is not universal among elderly individuals^[54–56]^. Data indicate that only a subset of elderly individuals will exhibit large clones, while others maintain relatively stable mutant clones that are relatively small. Additionally, CH carriers demonstrate remarkable variability in the development of diseases associated with this condition, implying the presence of additional factors that contribute to the distinct disease profiles observed within the population. Hence, it is crucial to unravel these underlying factors to gain a comprehensive understanding of the mechanisms involved. Such research endeavors may hold significance in optimizing clinical management strategies and potentially lead to the mitigation of the adverse outcomes associated with CH.

## FACTORS THAT PROMOTE CH

### Aging: age-related clonal hematopoiesis

The association between CH and aging is widely recognized. Numerous studies have been undertaken to characterize the association between age and pre-leukemic stem cells^[57]^, leading to the conceptualization of age-related clonal hematopoiesis (ARCH)^[8,9,15]^. Early studies demonstrated a positive correlation between the prevalence of CH and advancing age^[8,9,15]^, including studies that first deduced CH from analyses of skewing of X chromosome inactivation in women^[58]^. While individuals below the age of 40 showed little or no detectable CH, clones derived from mutations in candidate driver genes were observable in about 10% of individuals over 70 years of age^[8]^. The most commonly identified mutations were found in genes encoding epigenetic modifiers, such as DNA methyltransferase three alpha (*DNMT3A*) and ten-eleven translocation 2 (*TET2*). Since these initial reports, alternative methods of assessing clonality in blood samples have been employed. For instance, a study by Zink *et al.* utilized a non-biased, barcoded whole-genome DNA sequence analysis, which detected clonal events in 50% of individuals by the age of 85, indicating that CH was nearly an inevitable consequence of advanced age^[16]^. Surprisingly, in this study, only a small portion (less than 15%) of the clonal events could be attributed to mutations in known driver gene candidates. Similar conclusions have been reached by other studies employing non-biased analyses of CH^[59]^.

Related to the concept of age-related clone expansion, the application of ultra-deep, error-corrected sequencing to assess driver gene candidates has provided additional insights. These studies have revealed that small VAF clones (< 0.1%) are essentially ubiquitous by middle age, indicating that CH is prevalent in the general population during middle adulthood, and that clones expand with advanced age. The underlying mechanisms by which aging contributes to clone expansion in CH remain incompletely understood and require further investigation. Aging can be conceptualized as a multifaceted stressor that exerts diverse effects on the hematopoietic system. Mechanistically, this process involves a complex interplay of various factors, including but not limited to inflammation, cellular senescence, epigenetic reprogramming, metabolic alterations, and loss of clonality that may perturb the bone marrow niche and favor the expansion of mutant HSCs^[59–62]^. However, the interconnectedness of these factors makes it challenging to pinpoint a singular cause for clone expansion, particularly in the context of aging. Thus, it is imperative to delve into each factor individually to gain a better understanding of their specific impact on HSCs and their potential contribution to clone expansion. In the subsequent sections (see Sections “Chemotherapy and radiation: therapy-related clonal hematopoiesis (t-CH)-Genetic risk”), we will discuss these factors individually.

### Chemotherapy and radiation: therapy-related clonal hematopoiesis (t-CH)

As previously mentioned, while CH is closely associated with aging, external factors can accelerate the expansion of mutant clones within the hematopoietic system. In this context, therapy-related CH (t-CH) has emerged as a distinct form of CH frequently observed in individuals with a history of cancer or those who have undergone cancer treatment. Multiple studies have demonstrated an increased prevalence of t-CH among cancer survivors, with a substantial proportion exhibiting somatic mutations in tumor protein p53 (*TP53*) and protein phosphatase Mg2+/Mn2+-dependent 1D (*PPM1D*) genes, both of which are vital components of the DNA damage response (DDR) pathway^[63–65]^.

In an investigation led by Coombs *et al.*, a cohort of 8,810 non-hematological cancer patients underwent whole-exome sequencing of tumor tissue and blood^[63]^. The study revealed that approximately 24% of the patients harbored CH mutations, predominantly *TP53* missense mutations, and protein-truncating mutations in exon 6 of *PPM1D*, which result in a gain of function. The presence of these specific t-CH mutant clones was significantly associated with prior exposure to chemotherapy and radiation therapy, while the presence of other CH mutations generally did not show an association with chemotherapy exposure. Furthermore, the study demonstrated that the presence of t-CH increased the risk of developing subsequent hematological cancers and adversely affected overall survival, with most deaths attributed to the progression of the primary non-hematologic cancer^[63]^.

These findings were further supported by Wong *et al*., who used an error-corrected DNA sequencing method to investigate the impact of cytotoxic therapy-induced external stress on the expansion of t-CH clones^[64]^. Their cohort consisted of hematological malignancy patients with and without cytotoxic therapy exposure and 19 healthy donors. Similar to the previous study, patients who received chemotherapy and/or radiation exhibited a higher prevalence of CH clones, and *TP53* and *PPM1D* variants were enriched following cytotoxic therapy^[64]^. Additionally, Bolton *et al.* expanded upon these findings by analyzing a larger cohort of 24,146 patients with various primary tumor types. Again, the presence of cytotoxic therapy was associated with a greater likelihood of harboring t-CH gene mutations, with distinct gene-treatment effects and dose-response relationships observed^[65]^. Collectively, these studies indicate that t-CH is prevalent among individuals previously treated for cancer and suggest that cytotoxic therapy-induced genotoxic stress can confer selective pressure on these t-CH mutant clones.

The expansion of t-CH clones after cytotoxic therapy can be ascribed to multiple underlying factors. Available evidence suggests that t-CH clones primarily expand following therapy, rather than arising as *de novo* mutations^[66,67]^. Chemotherapy and radiation can inflict stress upon normal cells, including HSCs in the bone marrow, responsible for blood cell production. The DNA damage induced by these treatments can trigger senescence and apoptosis in wild-type HSCs, yet the HSCs with mutations in the DDR pathway are relatively resistant to these stresses. HSCs carrying these mutations can endure the stress through a type of cellular competition that has the potential to result in selective clonal expansion of the t-CH clones^[68]^. It also could be speculated that cytotoxic therapy creates an environment that facilitates the proliferation of t-CH clones. For instance, studies indicate that cytotoxic therapies can lead to changes in inflammatory processes and perturb the production of growth factors and cytokines^[69–74]^, which could conceivably confer a selective advantage for the expansion of mutated HSCs by facilitating their survival and/or proliferation. This concept will be discussed in a subsequent section.

In addition to their use in solid tumor therapy, chemotherapy and radiation play a crucial role in the management of various bone marrow disorders, transplantation procedures, immunodeficiencies, and autoimmune diseases. These treatments serve as cytotoxic conditioning regimens, creating suitable environments for engraftment, preventing rejection of transplanted cells or tissues, or suppressing an overactive immune response^[1,60]^. Therefore, further research is necessary to unravel the molecular mechanisms that underlie the expansion of t-CH clones following therapy, including the interplay between DDR pathways, clonal selection, and therapy-specific factors.

Related to the research conducted with cancer-related genotoxic stress, a number of laboratories are investigating the impact of space radiation on CH. This line of inquiry is particularly relevant in light of the expanding possibilities for space travel and the necessity to understand the potential health risks associated with prolonged exposure to space radiation, particularly during travels that extend beyond the confines of low-Earth orbit that will expose astronauts to powerful galactic cosmic rays and solar particle events. An investigation into the long-term effects of extended space travel in low orbit was studied by the National Aeronautics and Space Administration (NASA) Twin Study^[75]^. The study involved monitoring identical twins, denoted as HR (control subject with previous experience in short space flights) and TW (astronaut who had undergone 1 year of space travel aboard the International Space Station), both of whom were 50 years of age at the initiation of TW’s prolonged space travel. One finding from the study was the identification of genotoxic stress, elevated DNA damage responses, and genomic instability in leukocytes from prolonged space travel. When examining the CH profiles of both twins, it was observed that both HR and TW harbored CH mutations^[76]^. HR exhibited two *DNMT3A* mutations associated with a dominant-negative function, originating from different alleles. TW was diagnosed with a *TET2* mutation, which showed an increasing trajectory after completing his 1-year mission. While the study was limited to two subjects, the findings suggest that radiation exposure during space travel may contribute to CH clone growth and could potentially increase health risks.

CH mutations were also investigated in astronauts who had participated in relatively short Space Shuttle missions (median duration of 12 days)^[77]^. The median age of the Shuttle mission crew was approximately 42 years. Using ultra-deep, error-corrected DNA sequencing, 17 variants in 14 astronauts were detected using a myeloid panel targeting 37 frequently mutated genes. Among the 17 variants, the most commonly mutated gene was *TP53*, with seven different variants identified in six astronauts. This was followed by *DNMT3A*, with six variants detected in five astronauts. Together, these two genes accounted for 38% of the detected mutations. It is worth noting that the majority of observed mutations were small missense mutations (ranging from 0.10%-0.95%), well below the 2% threshold for a CHIP designation. While the pattern of *TP53* mutations resembles t-CH, raising the compelling prospect that space radiation may play a role in driving clonal selection, further studies are required to assess whether these clones will undergo further expansion over time. Additionally, both studies mentioned above examined individuals exposed to low-Earth orbit, and future analyses of astronauts exposed to deep-space radiation will be particularly significant.

### Inflammation

Inflammation has emerged as a potential factor in the development and progression of CH. Mounting evidence suggests that the chronic low-grade inflammation commonly observed in aging individuals, known as inflammaging, creates a proinflammatory microenvironment within the bone marrow niche, thereby promoting the expansion of mutant clones^[78–84]^. Inflammatory cytokines, such as interleukin-1 (IL-1), interleukin-6 (IL-6), and tumor necrosis factor-alpha (TNF-α), have been implicated in driving clonal selection and expansion by modulating the proliferation and self-renewal capacity of HSCs^[78–80,82]^. Importantly, the findings indicate that the interaction between inflammation and CH is bidirectional, as expanded mutant clones can also contribute to sustained inflammation through the production of proinflammatory factors^[79,84–86]^. Therefore, understanding the intricate relationship between inflammation and CH may be essential for unraveling the mechanisms underlying the development and progression of age-related diseases associated with CH. In this section, we discuss the experimental progress that has contributed to our understanding of CH-clone expansion and its association with age-related diseases, with a specific focus on the role of inflammation. The experimental and clinical studies reviewed in this section will focus on the commonly identified driver genes *TET2* and *DNMT3A* that are commonly mutated in CH.

#### TET2

Extensive research has elucidated the role of *TET2* in regulating HSC self-renewal and proliferation, providing mutant clones with a distinct competitive advantage over wild-type counterparts, and myeloid cell differentiation^[87–89]^. While the impact of inflammatory stress on TET2-mediated hematopoiesis has been explored^[78–82,85,87–90]^, the primary objective of this review will be directed towards elucidating the involvement of inflammation in the context of CH.

In 2017, a causal connection between *TET2*-CH and CVD was established through the induction of inflammation^[31]^. In this study, a competitive bone marrow transplantation (BMT) approach was used to mimic the expansion of hematopoietic clones observed in humans. Atherosclerosis-prone mice lacking the low-density lipoprotein receptor (LDLR) were engrafted with either 10% *Tet2*^+/+^ or *Tet2*^−/−^ bone marrow cells and subjected to a Western diet. Over time, *Tet2*^−/−^ cells gradually expanded with a slight skewing towards the myeloid lineage, leading to the progression of atherosclerosis and increased plaque size. *Tet2*^−/−^ macrophages were found to contribute to the exacerbation of atherosclerotic pathology through the increased secretion of inflammatory mediators, including interleukin-1β (IL-1β) and IL-6, and components of the nucleotide-binding oligomerization domain, leucine-rich repeat and pyrin domain-containing protein 3 (NLRP3) inflammasome via a histone deacetylase (HDAC)-dependent mechanism. Notably, treatment with an inhibitor of NLRP3 (MCC950) resulted in a reduction in plaque size^[31]^. A subsequent study used a conventional BMT method and confirmed that the transplantation of 100% *Tet2*^+/+^ or *Tet2*^−/−^ mouse bone marrow cells also led to increased atherosclerotic plaque size, accompanied by upregulated inflammatory cytokines and chemokines by *Tet2*^−/−^ macrophages^[18]^. These findings provided early documentation of the association between CH, inflammatory pathways, and atherosclerosis.

The findings discussed above were corroborated and extended in models of heart failure. The pathological and inflammatory effects of *TET2*-CH were demonstrated in models of pressure overload-induced cardiac hypertrophy through transverse aortic constriction (TAC) and by myocardial infarction induced by left anterior descending artery (LAD) ligation. Both models revealed the contribution of *Tet2*^−/−^ macrophages in NLRP3-mediated IL-1β production and the reversal of the cardiac phenotype upon administration of an NLRP3 inhibitor^[32]^. Moreover, experiments utilizing CRISPR/Cas9-mediated *TET2*-CH in a mouse model of cardiac dysfunction induced by angiotensin II infusion, along with a non-conditioned adoptive transfer model representing naturally occurring cardiac dysfunction in *TET2*-CH, corroborated the presence of inflammatory signatures^[33,91]^. Taken together, *TET2*-CH leads to increased activation of IL-1β signaling and likely other cytokines, which in turn contributes to the progression of CVD.

In accordance with the experimental findings, clinical studies have documented the presence of elevated inflammatory markers, such as IL-1β and IL-6, in individuals who carry *TET2*-CH mutations^[92]^. In addition, post-hoc analysis of the Canakinumab Anti-inflammatory Thrombosis Outcomes Study (CANTOS) trial indicated that individuals without detectable CH exhibited a 7% relative risk reduction in major adverse cardiovascular events (MACE) in response to any dose of canakinumab (a neutralizing IL-1β antibody). In contrast, *TET2*-CH displayed a remarkable 62% relative risk reduction in MACE^[93]^. These clinical findings are not only consistent with experimental studies in mouse CVD models, but also suggest that assessing CH in the patient population may provide a potential strategy to introduce precision medicine approaches for the treatment of CVD.

While investigations using mouse models and clinical data have provided compelling evidence for a causal relationship between *TET2*-CH and CVD, a recent investigation has introduced the concept of reverse causality, proposing that atherosclerosis may accelerate the expansion of CH by promoting the proliferation of HSCs^[94]^. This hypothesis is based on a combined analysis of mathematical modeling and experimental validation using a conventional BMT in a mouse model of atherosclerosis, comparing mice fed a control diet with those on an atherogenic diet. The results indicated accelerated *Tet2*^−/−^ clone growth in HSCs and myeloid cells under conditions of an atherogenic diet, suggesting a potential vicious cycle wherein atherosclerosis triggers the development of CH, which, in turn, drives further progression of atherosclerosis^[94]^. While these results are intriguing, it should be noted that prior experimental studies with *Tet2* had failed to find evidence for a reverse causality mechanism in models of atherosclerosis^[31,85,95]^, heart failure^[32]^, and diet-induced obesity^[96]^.

Another study focused on inflammatory stress induced by lipopolysaccharide (LPS) on the self-renewal capacity of hematopoietic *Tet2*-mutant cells^[79]^. Researchers used a conventional BMT model with pre- and post-LPS challenges. Analysis of peripheral blood samples revealed a significant reduction in the donor cell chimerism in control mice, indicating impaired repopulating capability. In contrast, the *TET2*-CH model exhibited no changes in its repopulating ability. This resilience of *Tet2*-mutant cells was attributed to increased cytokine expression, specifically IL-6. Additionally, reduced apoptosis in Lin^−^ and LSK cells in response to acute LPS challenge was demonstrated. The abnormalities induced by LPS could be reversed through the use of pharmacologic inhibitors targeting downstream regulators of the IL-6 signaling pathways: i.e., SH2 containing protein tyrosine phosphatase-2 (SHP2) or Signal transducer and activator of transcription 3 (STAT3), myeloid RNA regulator of Bcl-2-like protein 11 (BCL2L11)-induced cell death, and myeloid RNA regulator of Bim-induced death (Morrbid)^[79]^.

#### DNMT3A

*DNMT3A* stands out as the most frequently mutated CH driver gene in the elderly^[8,9,15]^. Loss of *DNMT3A* function in HSCs promotes self-renewal at the expense of differentiation, conferring a competitive advantage over normal HSCs^[97]^. Despite the contrasting enzymatic activities of *DNMT3A* and *TET2* in the regulation of DNA methylation, CH due to mutations in either gene has been implicated in the development of atherosclerosis and cardiac dysfunction through the activation of IL-1β/IL-6 pathway (with IL-6 being a downstream molecule of IL-1β)^[31,32,91,98]^.

Experimental studies indicate that *DNMT3A*-CH can expand in response to chronic infection via the interferon-gamma (IFN-γ) signaling pathway^[84]^. To investigate the expansion of *DNMT3A*-CH clones mediated by inflammation, a competitive BMT model using *Dnmt3a*^−/−^ was employed in conjunction with chronic *Mycobacterium avium* infection, an intracellular pathogen known to induce an IFN-γ-mediated immune response^[84]^. This phenomenon was additionally supported by demonstrating that administering recombinant IFN-γ was sufficient to trigger the expansion of *DNMT3A*-CH clone. Furthermore, the study showed that inflammation-mediated *Dnmt3a* clone expansion was associated with reduced differentiation and increased self-renewal. Interestingly, chronic infection did not result in elevated levels of IL-1β, IL-6, and tumor necrosis factor-alpha (TNF-α). Moreover, a study involving ulcerative colitis patients found higher levels of IFN-γ in their serum, especially among those with *DNMT3A*-CH, implying a potential link between *DNMT3A*-CH and IFN-γ^[48]^. Subsequent research extended these findings by revealing that IFN-γ signaling is crucial for *Dnmt3a*-mutant clone expansion in a mouse model^[99]^. This mechanism involves p53 stabilization and subsequent upregulation of p21. This IFN-γ-mediated CH selection was unique to *Dnmt3a* mutant clones, as *Tet2* mutants did not exhibit the same behavior. As discussed earlier, *TET2*-CH has been more associated with IL-1 and IL-6 signaling, particularly in response to microbial products such as bacterial lipopolysaccharide, endotoxin shock, or colitis^[31,79,90]^.

#### Clinical trials with anti-inflammatory agents

Numerous inquiries underscore a correlation between elevated proinflammatory cytokine levels and the expansion of CH, suggesting that mitigating or reversing disease progression could be achievable through the blockade of these inflammatory markers. Consequently, it could be hypothesized that individuals harboring CH clones may stand to benefit from anti-inflammatory interventions. Currently, three registered clinical trials are oriented towards addressing CH within the context of cardiovascular diseases. The first of these trials is a phase I study involving selnoflast, an NLRP3 inhibitor, designed to be administered to patients with *TET2*-CH and concurrent coronary artery disease (ISRCTN10520571). The second is a phase II study centered on colchicine, designed to be administered to individuals with CH and chronic heart failure (EudraCT 2021-001508-13). Lastly, the third trial involves DFV890 (an NLRP3 inhibitor) and MAS825 (an anti-IL-1β/IL-18 agent), targeting patients with CH and coronary heart disease (NCT06097663). However, to draw definitive conclusions regarding the efficacy of anti-inflammatory therapies in the context of CH, large randomized studies will be imperative. Furthermore, a pivotal consideration arises concerning the capacity of anti-inflammatory therapies to regulate clone size. The prospect of discerning the feasibility of implementing this strategy in carriers of CH, with preexisting associated pathologies, remains an open question.

#### The role of ROS

In addition to specific inflammatory mediators, HSCs may intricately engage with the age-associated inflammatory microenvironment^[100,101]^. Factors such as DNA damage and replication stress, alterations in epigenetic profiles, and diminished autophagic activity contribute to the accumulation of mitochondrial stress and an augmented production of reactive oxygen species (ROS)^[102–105]^. For instance, a study has demonstrated the instrumental role of *DNMT3A* and *TET2* in maintaining mitochondrial DNA integrity. The loss of function of these genes resulted in the activation of cyclic GMP-AMP synthase (cGAS) signaling and type I interferon pathway, indicating a potential association between mitochondrial dysfunction, inflammation, and CH^[106]^. Furthermore, macrophages with *Ppm1d* mutations exhibited an impaired DNA damage response pathway, leading to increased production of ROS and IL-1β in response to lipopolysaccharide (LPS). The application of hydroxy-2,2,6,6-tetramethylpiperidin-1-oxyl (TEMPOL), a ROS scavenger, rescued the elevated levels of ROS and IL-1β, suggesting an interplay between ROS, inflammation and CH^[34]^. Despite the significance of these two findings, it is important to note that both studies were conducted *in vitro* using monocyte-derived macrophages, thereby deviating from the *in vivo* context of the CH phenomenon and lacking a pathological tissue environment. Therefore, the precise causal relationship between these determinants and CH necessitates further comprehensive investigative scrutiny in a physiological system.

### Obesity/metabolic dysfunction/diet

Obesity, metabolic dysfunction, and an unhealthy diet have been associated with chronic low-grade inflammation, fatty bone marrow, dyslipidemia, insulin resistance or hyperglycemia, and metabolic imbalances^[107]^. These metabolic abnormalities can disrupt the function of HSCs through the perturbation of metabolic homeostasis^[108–110]^. In the context of obesity, the inflammatory state can compromise the HSC niche, impairing the signaling environment necessary for proper HSC maintenance and self-renewal^[109]^. Therefore, understanding the influence of metabolic factors on CH and the complex interrelationship between metabolism and HSC biology is of significant interest.

Studies have demonstrated associations between CH and an increased risk of type 2 diabetes mellitus (T2DM). Even after adjusting for potential confounding factors, individuals with T2DM are more likely to be CH carriers^[8,111,112]^. Obesity has also been shown to have an impact on CH^[40]^. Pasupuleti *et al.* investigated the association between obesity and CH through whole-exome sequencing, using data from 47,466 individuals^[40]^. The analysis of clinical data revealed that CH was present in 5.8% of the study population and was strongly associated with a significant increase in waist-to-hip ratio, indicating that obesity may promote the expansion of CH clones or *vice versa*.

On the other hand, inconsistencies exist in the clinical data regarding the relationship between CH and metabolic stress. Kar *et al.* identified individuals with CH using whole-exome sequencing of 200,453 UK biobank participants and observed a higher genetically predicted body mass index (BMI) in individuals with larger CH clones and an increased presence of circulating apolipoprotein B in those with *TET2*-CH clones^[26]^. However, no significant association was found between obesity or T2DM and CH^[26]^. In a larger cohort study involving 454,803 participants from the UK Biobank and 173,585 participants from the Geisinger MyCode Community Health Initiative (GHS), Kessler *et al*. reported different results^[27]^. They found a negative association between BMI and fat percentage with *DNMT3A*-CH mutant clones, while other CH mutations, including *TET2*, exhibited a positive association, suggesting that the relationship may be gene-specific. Moreover, an exploratory study compared 1,050 obese individuals who received standard care to those who underwent bariatric surgery (a weight-loss surgery aimed at improving metabolic status) and tracked their progress for 20 years^[113]^. Although similar clone sizes were shown between obese individuals treated with usual care and those with bariatric surgery, a significant association was found between clone size and age only in the usual care group, but not in the bariatric surgery group. Trajectory analysis revealed that obesity had associations with clone growth, insulin resistance, and low levels of high-density lipoprotein cholesterol (HDL-C), suggesting that dysfunctional metabolic stress might contribute to clone growth or *vice versa*.

Finally, in the UK Biobank cohort, a higher prevalence of CH was observed in individuals with poorer diet quality, characterized by increased consumption of red meat, processed food, and added salt^[114]^. These findings were supported by a study involving 8,709 postmenopausal women who were free of cancer or CVD, which demonstrated a significant association between maintaining a normal BMI and a lower prevalence of CH, underscoring the potential impact of metabolic factors on the development of CH^[115]^.

As noted above, human intervention studies have yielded mixed results, and issues of directionality and causality cannot be discerned. These findings indicate the need for further research to establish the mechanistic relationships between interventions targeting metabolic control and their potential influence on CH. Consequently, several animal studies have been conducted to investigate the relationship between CH, obesity, and T2DM. Fuster *et al*. conducted a study using a mouse model of *Tet2*-mutant CH^[96]^. This mouse model exhibited insulin resistance and hyperglycemia in both naturally aged mice and obese mice induced by a high-fat and high-sucrose diet, without significant changes in body weight or fat mass compared to the control (non-CH) group. The underlying mechanism was attributed to an elevated level of NLRP3 inflammasome/IL-1β-mediated inflammation. In another study, it was reported that mouse models of CH carrying mutations in *Tet2, Dnmt3a, Asxl1,* and *Jak2* demonstrated that obesity accelerated the size of CH clones and increased the risk of developing a myeloproliferative neoplasm (MPN)-like phenotype, regardless of the specific mutation^[40]^. Furthermore, this study also found that the *Tet2*-CH mice also exhibited a phenotype of hyperglycemia and elevated levels of proinflammatory cytokines, including IL-6, IL-1β, and TNF-α, compared to the control group. If confirmed with additional data, these results would suggest a role of CH exacerbating obesity-induced metabolic dysfunction.

Lastly, an experimental study examined the impact of bone marrow adiposity on a model of *Dnmt3a*-CH mutant mice^[116]^. Bone marrow adiposity serves as an indicator of natural aging, and obese individuals tend to have a higher proportion of fat within their bone marrow due to adipocyte expansion^[117,118]^. Thus, bone marrow adiposity can potentially alter hematopoiesis. Using a NOD-SCID-Gamma (NSG) mouse model, which allows the transplantation of both human and mouse HSCs, the investigators found that an elevated level of bone marrow adipogenesis was associated with increased mutant clone size compared to the control group^[116]^. Furthermore, the growth advantage of the mutant clone was more pronounced when aged mutant donor cells were transplanted. This phenomenon was also associated with the upregulation of inflammatory pathways (IFN-α, IFN-γ, TNF-α, and IL-6), and clone growth was reversed by the administration of the neutralizing IL-6 antibodies. This insight holds promise for further understanding and potentially manipulating the dynamics of CH in the context of aging and obesity, offering potential avenues for future therapeutic interventions in related medical conditions.

### Lifestyle

Related to the considerations discussed in the section above, lifespan and lifestyle factors are intimately intertwined. Given that CH is closely associated with advancing age, it is of interest to understand the associations between CH and lifestyle factors. Various lifestyle aspects have demonstrated robust associations with CH^[62]^. These lifestyle factors include tobacco use or smoking^[9,16,26,27,63,65,119]^, sleep deprivation^[85]^, psychiatric stress^[16]^, factors that impact the epigenetic clock^[50,51]^, early menopause^[20]^, and vitamin C deficiency^[120]^. Thus, it seems reasonable to conclude that by embracing a healthy lifestyle, individuals can diminish their likelihood of developing CH and thereby positively impact their healthspan and lifespan.

### Genetic risk

The preceding paragraphs have primarily addressed the effects of extrinsic stressors on CH. However, a series of studies have shed light on the significant influence of inherited germline variants on CH clones. Although the impact of heritable genetics on CH development is relatively modest compared to the risk of advanced age, collaborative efforts of large cohorts have identified specific loci that influence the likelihood of CH clone expansion.

Initial genome-wide association studies (GWAS) identified telomerase reverse transcriptase (*TERT*) locus associated with CH^[16]^. This pioneering work was subsequently expanded by Bick *et al*., who employed whole-exome sequencing techniques to investigate a vast cohort of 97,691 participants from diverse ancestral backgrounds within the Trans-omics for Precision Medicine (TOPMed) program^[92]^. It was observed that individuals carrying the *TERT* variant allele exhibit a 1.3-fold higher risk of developing CH, presumably due to an elevated susceptibility to acquiring mutations from the impairment of maintaining genome integrity. While *TERT* is the most significantly associated germline locus for CH in GWAS, subsequent investigations identified a relationship between CH, leukocyte telomere length (LTL), and CAD^[121]^. Bidirectional Mendelian randomization analysis of data from the TOPMed and UK Biobank cohorts indicated that longer genetically imputed LTL contributes to an increased propensity to develop CH^[121]^. It was also observed that CH, in turn, accelerates the shortening of measured LTL^[121]^. One potential explanation is that elongated telomeres provide cells with increased cellular longevity, thereby extending the temporal window during which mutational events may occur. Upon acquisition of a CH driver mutation, the increased proliferation and cell divisions may expedite the rate of telomere shortening. Subsequently, a comprehensive analysis of the UK Biobank dataset comprising 200,453 participants replicated the findings, confirming the association between CH and the *TERT* locus, specifically in populations of European ancestry^[26]^.

Additional analyses of cohorts have enabled the identification of additional genetic variants that predispose to CH. For example, Bick *et al*. uncovered genetic loci located within the intergenic region spanning Karyopherin Subunit Alpha 4/Tripartite Motif-Containing Protein 59 (*KPNA4/TRIM59*), and another genetic variant, prevalent in individuals of African ancestry, was identified near the *TET2* locus^[92]^. These two variants were associated with a 1.16-fold and 2.4-fold increased risk of developing CH, respectively^[92]^. Further characterization of the variant located near the *TET2* locus indicated that it disrupts a distal enhancer.

Another study identified 10 novel genetic loci that exhibited significant associations with CH^[26]^. These loci were implicated in processes such as DNA damage, oncogene signaling, telomere maintenance, and blood cell homing, highlighting their potential contributions to CH development. Interestingly, the study identified a distinct germline variant at T-cell leukemia/lymphoma protein 1A (*TCL1A*) that exhibited an opposing impact on *DNMT3A* and *TET2*, emphasizing the intricate interplay between germline genetic factors and the manifestation of CH. This observation was supported by the study of Kessler *et al.,* which analyzed whole-exome sequencing data from an extensive cohort of 628,388 individuals in the UKB/GHS^[27]^. Their findings provided additional evidence by demonstrating an augmented risk of *DNMT3A-*CH but a reduced risk of *TET2*-CH by the *TCL1A* variant, and they identified an additional 24 germline variant loci predisposing individuals to CH.

The potential explanation for the diverse impact observed between the CH driver gene and *TCL1A* has been recently illustrated by the analysis of “passenger-approximated clonal expansion rate (PACER)”, a method that enables the estimation of mutation fitness^[122]^. Using this method, it was found that the presence of the common *TCL1A* variant leads to a slower expansion of *TET2* and other CH mutant clones, but has little effect on the expansion of *DNMT3A* mutant clones. In alignment with the PACER prediction, they observed that the *TCL1A* variant was associated with a 4% reduction in the expansion of *TET2* mutant clones. This observation was made in the Women’s Health Initiative dataset, where targeted sequencing was conducted across two time points with an average duration of 16.2 years. Subsequent exploration revealed that when driver mutations, including *TET2* and others (excluding *DNMT3A*), are present, excessive *TCL1A* expression occurs, thus promoting clonal expansion. Conversely, the *TCL1A* variant, even in the presence of CH-associated mutations, restricts chromatin accessibility, leading to reduced expression of *TCL1A* and abrogates the clonal advantage^[122]^. Thus, by uncovering loci that predispose to CH, it may be possible to identify potential therapeutic targets (e.g., *TCL1A*) and develop strategies to intervene in the early stages of diseases that are promoted by CH.

## CONCLUSIONS

CH appears to represent an inevitable consequence of the aging process. Clone expansion in the hematopoietic system is intricately associated with a multitude of regulating factors, including chemotherapy/radiation, inflammation, metabolic stress, lifestyle choices, and genetic predisposition [Figure 1]. The cumulative impact of these interconnected factors is increasingly recognized to impact the process of biological aging and increase vulnerability to diverse age-related diseases and mortality. Despite the advancements summarized herein, many questions remain unanswered regarding the detailed underlying mechanisms governing clone expansion in CH and its subsequent ramifications for age-related diseases. Additional explorations of regulators of clone expansion will contribute to a deeper understanding of age-related diseases and aging *per se*. At this early stage, it is reasonable to speculate that the identification of CH in the patient population will pave the way for the development of targeted therapeutic strategies directed towards age-related diseases where inflammation plays a key role.
